# The relationship between forests and freshwater fish consumption in rural Nigeria

**DOI:** 10.1371/journal.pone.0218038

**Published:** 2019-06-11

**Authors:** Michaela Lo, Sari Narulita, Amy Ickowitz

**Affiliations:** Center for International Forestry Research (CIFOR), Bogor, Indonesia; Sveriges landbruksuniversitet - Campus Umea, SWEDEN

## Abstract

Nigerians depend on fish for maintaining diverse and healthy diets. Fish are a key source of protein and micronutrients, both of which are important for healthy diets. Some research has shown that forests provide important ecosystem functions that support the productive capacity and sustainability of inland fisheries. Our study aims to empirically assess the relationship between forest cover around rivers and fish consumption. We use data from the Living Standards Measurement Survey (LSMS) and spatially merge household and village data with forest cover and river maps. We estimate the relationship between forest cover around rivers and average village fresh fish consumption, while also accounting for other socio-economic and geographical determinants. We find that that the density of forest cover around rivers is positively and significantly correlated with village consumption of fresh fish. Our results suggest that forests influence the consumption of fresh fish by improving the productivity of inland fisheries and increasing the availability of fish. Aquatic habitats tend to be overlooked in debates on land use and food production, and yet can be critically important sources of nutrient-rich foods that are limited in rural diets in developing countries, particularly for the poor. Clearing forests for agriculture in order to produce more agricultural crops might have the unintended consequence of reducing another important food source.

## Introduction

Increasing agricultural land at the expense of forests has often been viewed as a necessary means to feed a hungry planet [1, 2]. While this is often lamented because of the impacts on biodiversity conservation and climate change [3–5], recent research has shown that deforestation may also have negative consequences on the diets of local communities that are part of the landscape where these land use changes occur [6]. While the direct provisioning that forests provide in the form of fruits, mushrooms, tubers, insects, and wild meat are increasingly recognized [7, 8]; there are other indirect pathways through which forests may impact diets. One key pathway is through the ecological functions that forests provide to wild fisheries given that fish can be a very important animal source food, particularly for poor communities in the tropics.

Several studies have found that forests contribute to sustaining the aquatic-terrestrial landscape and promote both the quality and quantity of freshwater fish populations [9–11]. Forests and riparian vegetation are beneficial in strengthening the biological integrity of aquatic ecosystems [12, 13] and support fish production in inland waters [10, 14]. Inland captured fisheries are important for supporting the diets of local communities who live near floodplains and rivers [15]; yet, these important contributions are poorly reported, and therefore globally undervalued [16, 17].

Fish are highly rich in protein and other key micro-nutrients such as Vitamin A, calcium, iron, and zinc [18]. Animal source foods are often absent or limited in the diets of the poor [19, 20] potentially resulting in malnutrition and a higher susceptibility to illnesses [21]. Fish are often the cheapest and most frequently consumed animal source food among the poor [22] and can make an important contribution to diversity in otherwise monotonous diets. They are also rich sources of fatty acids, which have shown to have significant importance for the growth and development of infants and young children [23].

The objective of this study is to empirically assess whether or not there is a relationship between forests and the amount of freshwater fish consumed in rural communities. We have two interlinked hypotheses that we believe underlie this relationship: First, that forested rivers increase the productivity of inland fisheries through the provisioning of terrestrial resources and regulatory mechanisms that support the functioning of freshwater ecosystems; and second, that the greater fish productivity of rivers with nearby forests results in higher fresh fish consumption. To test our hypotheses, we integrate data from the World Bank’s Living Standards Measurement Study-Integrated Surveys on Agriculture (LSMS-ISA) 2012–13 in Nigeria and forest distribution maps from Hansen, Potapov [24] at the village level. Nigeria is a large country with diverse eco-regions from the humid tropics in the south to dry and arid regions in the north. It is also home to many rivers, streams, and tributaries. This makes it an excellent country in which to test the hypothesis that forests have an indirect effect on peoples’ diets through their contribution to wild fisheries production. We run a series of robustness checks to test the validity of our results and their sensitivity to varying spatial extents of forest cover measures around rivers. We find that more fresh fish are consumed in villages with more forest cover bordering their rivers than in other villages with less forest cover after controlling for potential confounding variables.

### Forests and riverine and floodplain ecosystems

Forests provide several indirect functions that support riverine and floodplain ecosystems. First, the terrestrial environment determines the balance between organic inputs from aquatic sources and sources from vegetation fringing streams [12, 25]. Several studies have found that fish species that are highly dependent on terrestrial organic matter as a source of food, thrive in forested zones in comparison to other environments [12, 26, 27]. Second, terrestrial environments can alter the biophysical structure of aquatic bodies. Several studies found that forested sites contained larger materials with a more stable substrate composition than non-forested sites [25, 28, 29]. Iwata, Inoue [30] found that deforestation increased the input of fine sediments in lower reaches. Aquatic animals are sensitive to these biophysical changes and can suppress populations which depend on coarser substrates. Third, forests alongside water habitats help retain sediments and prevent erosion resulting in greater transparency of waters. This supports fish species that are more sensitive to turbidity [12, 31, 32] and those that are visually orientated [12, 33].

### Nigerian context

Nigeria has high rates of undernutrition; in 2015, 33% of children under five were stunted (NBS [34]). Stunting is usually associated with poor quality diets and insufficient micronutrient intake [35]. High levels of micronutrient deficiencies have been found across Nigeria, particularly in rural areas, which are constrained by poor access and unstable supplies of food [36–38]

In many Nigerian communities, fish, or fish products are considered a necessary element of the meal, either as the main source of protein, or as a condiment [39]. Fish are the preferred source of animal protein, perhaps due to its relative affordability, and are thus typically favored by low income households [40]. Fishing in communities that can directly harvest from aquatic bodies, particularly those which are open-access, are important sources of nutrients which contribute towards healthy and diverse diets (*ibid*.).

Nigerian forests cover around 9.6 million hectares of land [41]. Degraded forests and woodlands are the most dominant sub-class, followed by gallery forests which border the many streams and wetlands found mostly in the heart of the country. Nigeria’s forests face increasing threats from large-scale logging and conversion to farmlands. Between 2010 and 2015, the greatest annual net deforestation rate experienced in Africa was in Nigeria (410,000 ha/year) [42]. Riparian forests in particular, face growing pressures from urban expansion, which resulted in a 66% loss between 1984 and 2014 [43].

Around 900km^2^ of the country is covered in rivers and lakes supporting freshwater fish that are widely consumed by urban and rural households [44]. Most fish in Nigeria are sourced from artisanal fisheries found in rivers [39] contributing to 80% of domestic production [45]. There have as yet been no studies, to our knowledge, that investigate the relationship between vegetation cover and fish production or consumption in Nigeria.

## Data

### Fish consumption data

We use the 2012–2013 Nigerian LSMS-ISA data from the World Bank which has nationally representative data on a wide range of socio-economic variables for a sample of 4,697 households (NBS [46]). After limiting our sample to rural households only, we were left with 3,244 households. 42 households were excluded as data were missing or presented extreme outliers. Since our key variables of interest are relevant at the village scale, we aggregated household information to the Local Government Area (LGA) level (which will henceforth be referred to as villages for clarity) resulting in a sample of 309 villages. The LSMS reports quantities of food items consumed by the surveyed households for the week before the interview. Due to the perishability of fresh fish and the poor infrastructure in rural Nigeria, we hypothesize that fresh fish are more likely to be purchased, captured, and consumed locally rather than imported from long distances. Processed fish can also be sourced locally, but because of their higher preservability, they are able to travel long distances even in the absence of good infrastructure. Therefore, we do not include processed fish consumption in our main variable of interest and only focus on consumption of fresh fish. Instead, we investigate whether there is an association between forest cover and processed fish consumed (dried, smoked, and frozen fish) to check the robustness of our main results. Quantities of fresh fish measured in local units were converted into kilograms using food conversion units provided by the World Bank.

### Spatial data

**Fig 1:**
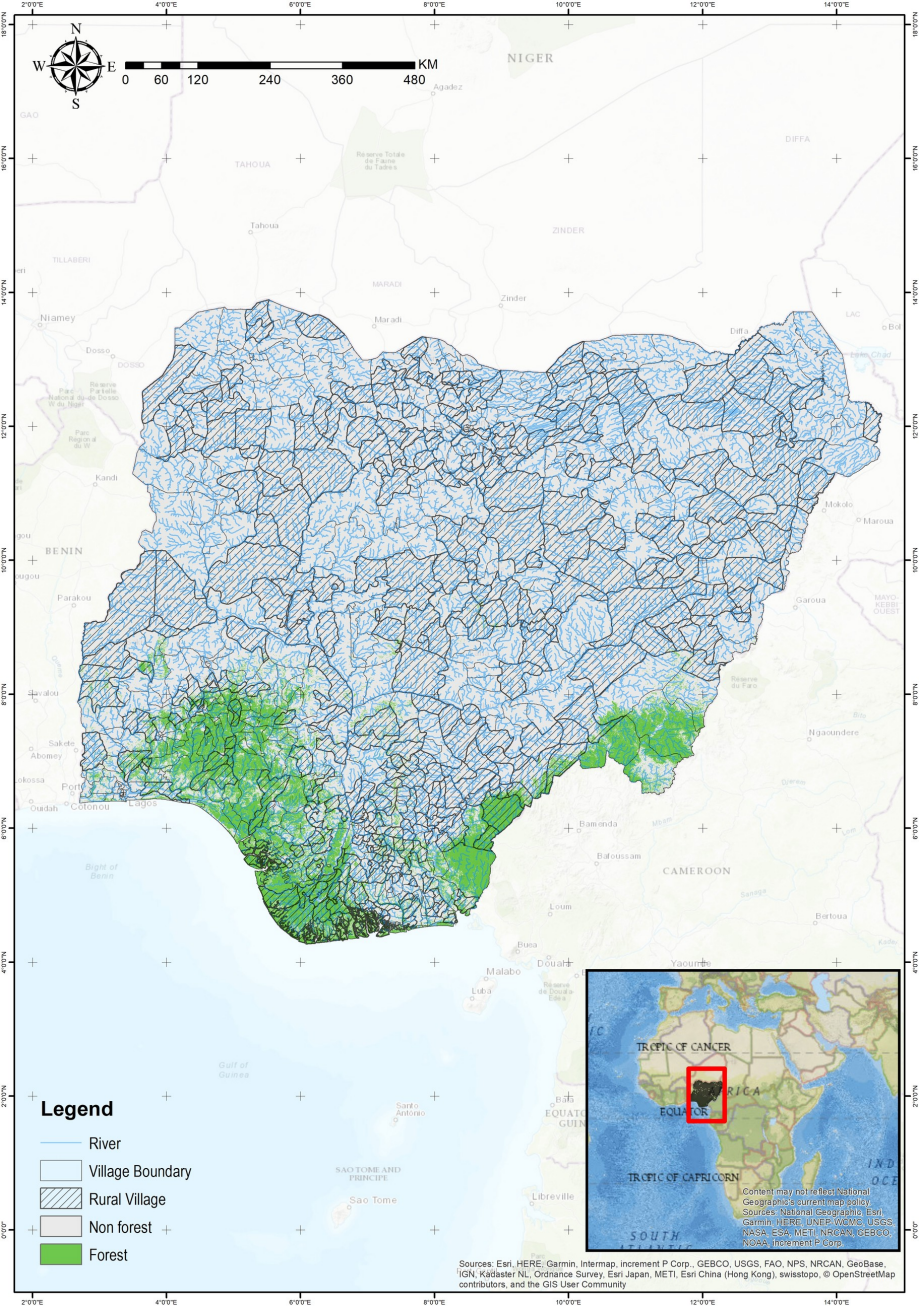
Map of Nigeria showing the location of rural villages (LGA) and the distribution of rivers and forest cover in 2012.

LGA administrative boundary information from the Global Administrative Area (GADM) [47] was used to spatially join data from LSMS with information on rivers and forest cover distribution across Nigeria at the village level. Village boundaries are shown in Fig 1. Data on river and lake maps came from the U.S. Geological Survey HydroSHEDS and HydroLAKES database, respectively [48, 49]. National forest cover for the year 2012 was calculated using the tree cover dataset provided by Hansen, Potapov [24]. Fig 1 shows the distribution of forest cover and rivers across Nigeria.

The Hansen dataset provides estimates for tree cover in the year 2000 at a 30 x 30 meter resolution with estimates of tree cover gain and loss in the following years up to 2017. To correspond with the LSMS data for the year 2012, we used this information to calculate forest cover in 2012. Tree cover is defined as vegetation higher than 5 meters from the ground [50]. We defined a pixel as ‘forest’ if it contains 30% or more tree cover and assign it a value of 1 (otherwise 0). If forest loss occurred between the 2000 and 2012 period, we reassigned the pixel to ‘non- forest’. If forest gain occurred after forest loss, we assigned pixel to forest. We then estimated forest cover density around rivers at the village level. The hydrological information from NASA’s Radar Topography Mission provides a high resolution of freshwater habitats at 15 arc-second for mapping stream networks [48]. We calculate forest cover around rivers that were within a 1km, 5km, 10km, and 20km radius around each village. Rivers close to the village are likely to be an accessible source of fresh fish, however, we also included larger buffers around villages as forest cover in uplands areas can impact fish communities further downstream [51–53]. In addition, this allows us to account for fish that may have been captured in rivers outside of the immediate village boundary, but still within relatively close proximity. We calculated the percentage of forest cover that is within river buffers of 100m, 500m, 1km and 2km to compare the influence of forest cover at different proximities to the river edge. Table 1 shows the definitions for each of the ten measures of forest cover that we use in the analysis.

**Table 1:** Forest cover variables measured around rivers according to the radius extension around each village (v) and river buffer width (r). We included rivers that were within 1km, 5km, 10km, and 20km of each village. Forest density was measured within buffers of 100m, 500m, 1km, and 2km around rivers.

		Radius around village (v)			
		1km	5km	10km	20km
**Buffer width around rivers (r)**	**100m**	r100v01 Forest cover measured within 1km radius extension of village and 100m around rivers	r100v05 Forest cover measured within 5km radius extension of village and 100m around rivers	r100v10 Forest cover measured within 10km radius extension of village and 100m around rivers	
	**500m**	r500v01 Forest cover measured within 1km radius extension of village and 500m around rivers	r500v05 Forest cover measured within 5km radius extension of village and 500m around rivers	r500v10 Forest cover measured within 10km radius extension of village and 500m around rivers	
	**1km**	r1kmv01 Forest cover measured within 1km radius extension of village and 1km around rivers	r1kmv05 Forest cover measured within 5km radius extension of village and 1km around rivers	r1kmv10 Forest cover measured within 10km radius extension of village and 1km around rivers	
	**2km**				r2kmv20 Forest cover measured within 20km radius extension of village and 2km around rivers

### Model

All spatial and household data are averaged to the village level for the analysis. Since a large part of the sample did not consume any fresh fish in the week preceding the interview, we use Cragg’s double hurdle model [54, 55] which enables us to partition the model into two parts that can be thought of as two stages in a decision process: 1) the decision of whether or not to consume any fish; and 2) for those who consume fish, how much fish to consume. The first stage is modelled as a binary decision, while the second stage models the quantity part of the decision after the ‘hurdle’ of whether or not to consume is passed. To account for the different factors that affect the probability of consuming fish and the amount consumed, we use separate equations for each stage of the model. The first stage for whether or not there is any fish consumed in a village is modelled by the following equation:

d=α+βp+γD+δZ+ωW+ε (1)

The second stage in which the dependent variable is a continuous measure of average quantity of fish consumed is modelled as:

Y=θ+ϑF+μH+ρB+πp+σD+τZ+φ (2)

where *d* is a dummy variable equal to one if there was any fresh fish consumption reported in the village in the week preceding the survey and zero otherwise; *p* represents the average price per kg of fish in the state in which the village is located; *D* is a vector of distance including: distance to market, distance to closest lake, and distance to coast which are included to control for access to fresh fish sources other than from rivers; *Z* represents elevation to account for altitudinal differences in fishery yields; W is a dummy variable indicating whether the village is located in the warm-humid region (categorized as tropic-warm/humid or tropic-warm/sub-humid agro-ecological zones in LSMS-ISA); and *ε* is an error term.

In Eq 2, *Y* represents the average quantity of fish consumed in the village in the week preceding the survey; *F* is a vector of forest cover around rivers; *H* is a vector of average household characteristics including: average household size, average age of household head, average education of household head, and average village wealth (estimated using principal component analysis on a subset of assets reported in the data); *B* is the average amount of beef consumed in the preceding week since fish is considered to be a substitute for meat in Nigeria [56, 57]; and *φ* is an error term.

As can be seen in Fig 1, most of Nigeria’s forests are located in the southern more humid zones so we run our models both on the entire sample as well as on the warm humid and sub-humid sample. We run the model separately for each of the ten measures of forest cover around rivers (Table 1) to test the sensitivity of our results to differences in spatial scale. We also run the same series of models with processed fish consumption (the sum of smoked, dry, and frozen fish) as the dependent variable; for these models, *d* represents presence or absence of consumption of any processed fish and *Y* represents the village average consumption of processed fish above zero.

## Results and discussion

Descriptive statistics for all the variables included in the model are presented in Table 2.

**Table 2:** Descriptive statistics including mean and standard deviation of dependent and independent variables for all villages and for villages in warm humid and warm sub-humid zones.

Variables	All villages			Villages in warm-humid zones		
	*N*	*Mean*	*Std*. *Dev*.	*N*	*Mean*	*Std*. *Dev*.
Av. household consumption of all types of fish (kg/week)	309	0.782	0.610	190	0.974	0.579
Av. household consumption of processed fish* (kg/week)	309	0.583	0.498	190	0.792	0.483
Av. household Consumption of fresh fish (kg/week)	309	0.199	0.339	190	0.182	0.317
Only villages that ate fresh fish (kg/week)	147	0.418	0.387	84	0.412	0.364
Av. household size	309	5.95	1.81	190	5.29	1.73
Av. age of household head (years)	309	51.97	7.30	190	53.96	7.72
Av. sex of household head (male = 1)	309	0.83	0.19	190	0.76	0.19
Av. education level of HH head**	309	1.76	0.59	190	1.92	0.56
Av. wealth index of household	309	-0.002	1.56	190	0.472	1.63
Av. household consumption of beef (kg/week)	309	0.44	0.42	190	0.42	0.36
Forest cover variables (%)ª						
r100v01	309	13.11	22.57	190	21.32	25.58
r100v05	309	12.93	21.58	190	21.02	24.25
r100v10	309	12.87	21.12	190	20.92	23.61
r500v01	309	13.00	22.83	190	21.14	26.01
r500v05	309	12.99	22.09	190	21.12	24.95
r500v10	309	13.00	21.75	190	21.13	24.46
r1kmv01	309	12.97	23.21	190	21.08	26.58
r1kmv05	309	13.00	22.54	190	21.14	25.59
r1kmv10	309	13.04	22.24	190	21.20	25.15
r2kmv20	309	13.21	21.94	190	21.47	24.62
Price of fresh fish (Naira/kg)	309	717.24	552.99	190	894.82	625.29
Elevation (m)	309	288.13	218.73	190	191.79	177.79
Distance to coast (km)	309	406.96	301.80	190	195.40	152.39
Distance to lake (km)	309	29.00	20.48	190	30.55	18.41
Distance to market (km)	309	69.84	38.53	190	76.30	39.58

*Total amount of frozen, dried, and smoked fish consumed

**Education of household head was categorized as follows: None = 1, Primary = 2, Secondary = 3, Higher = 4

ªRefer to Table 1 for descriptions of forest cover variables

The descriptive statistics in Table 2 show that overall, the average household consumes around 780g of fish within a week. Across all villages, households ate nearly three times more processed fish than fresh fish. For villages that consumed fresh fish (48%), the average intake was around 418g per household, however, due to the high number of households that did not consume fresh fish in the week before the survey, average fresh fish intake for all households sampled was just less than 200g.

Forest cover around rivers is around 13% across the whole sample and 21% in the warm humid and warm sub-humid zones; both remain fairly uniform across all of the different bands used in the analysis. This is because there are many villages with zero forest cover effectively flattening the overall average differences across the measures (between 21% and 37% of the village have zero forest cover depending on the). If we include only those villages with positive forest cover, then the mean forest cover ranges from 16.8% to 21% (See S1 Table). Average price of fish is around 700 Naira/kg and this varies widely, as seen by the large standard deviation.

We ran the model with different spatial buffers around villages and rivers. The coefficients for these buffers were quite similar in magnitude and statistical significance. We report below the model with best fit as determined by the pseudo-R^2^ with forest cover measured within 1km buffer widths of rivers and 1km buffer extensions of villages (*r1kmv01*). Results from the first stage of the model are presented in S2 Table. Table 3 presents the results from the second stage of the hurdle regression which models the average quantity of fresh fish consumed (in villages that consumed fresh fish) in the week before the survey across rural Nigeria as a function of forest cover around the rivers near the village and other control variables. The results of the main regression model are presented in Table 3, Column I; we see that forest cover around rivers near a village is positively associated with the quantity of fresh fish consumed. The marginal effect of forest cover on fish consumption is 0.00125 meaning that a 1% increase in forest cover would result in an increase of 1.25 average grams of fresh fish consumed; an increase in forest cover from its median value to the top 90^th^ percentile, would result in an increase of 54 grams or 27% more fresh fish consumed per week.

**Table 3:** Results from the second stage of the double hurdle model for fresh fish consumption and forest cover around rivers (*r1kmv01*) at the village level for all rural villages (n = 309) and the in warm-humid regions of Nigeria. Z-statistics are given in parentheses.

	Fresh fish consumption (All rural villages)	Fresh fish consumption (Warm-humid region only)
*2*^*nd*^ *Stage*	*I*	*II*
Forest cover	0.015*	0.015***
	(-1.94)	(-2.73)
Household size	0.093	-0.057
	(-1.02)	(-0.83)
Age of household head	-0.013	0.0005
	(-0.57)	(-0.03)
Education of household head	-0.152	0.064
	(-0.44)	(-0.22)
Wealth index of household	0.038	0.007
	(0.29)	(0.07)
Beef consumed by household	-0.138	-0.255
	(-0.42)	(-0.82)
Fresh fish price	0.0004	0.0004
	(-1.37)	(-0.60)
Distance to lake	-0.024**	-0.010
	(-2.15)	(-1.55)
Distance to market	-0.0004	-0.005
	(-0.13)	(-1.51)
Distance to coast	0.001	0.004**
	(-1.55)	(-2.27)
Elevation	-0.002*	-0.003**
	(-1.76)	(-2.25)
Constant	-0.235	0.148
	(-0.13)	(-0.11)
**Pseudo R**^**2**^	**0.0873**	**0.192**
**N**	**309**	**190**

*p<0.1

**p<0.05

***p<0.01

The results show that none of the household characteristics included in the model were significantly associated with the consumption of fresh fish. In particular, average wealth was not a significant determinant of fresh fish consumption, which suggests that fresh fish intake is similar between poorer and richer households. Contrary to results from Adah and Hope [56] that found fish to be a substitute for beef, our results show no significant association between fresh fish and beef consumption. After modelling average beef consumption as the dependent variable, we found that unlike fresh fish, wealth was a very important predictor of beef consumption which may indicate that fresh fish consumption is more opportunistic and less driven by market forces than other animal source foods in Nigeria (See S3 Table for results). Households that were closer to lakes consumed more fresh fish, however, the distance to the nearest key market had no statistically significant impact on fresh fish consumption. This could be attributed to the high perishability of fresh fish matched with the expensive transaction costs to deliver to key markets making fresh fish a food that is not significantly traded in large markets [58]. Greater fresh fish intake occurred at lower elevations, which could be linked to the fish being sourced from deltas and low level floodplains [59, 60].

As can be seen in Fig 1, forest cover has a high density in southern Nigeria with very little forest cover in the northern arid regions. We thus ran the models again restricting the sample to the humid and warm sub-humid zones. We report these results in Table 3, column II. The magnitude of the coefficient on forest cover is similar to the full sample, but the statistical association is stronger with confidence levels increasing from the 90% to 99% confidence level for the warm-humid sub-sample (See S4 Table for regression results across all measures of forest cover). Results also show a positive and significant relationship between fresh fish consumption and distance to coast. This suggests that in warm humid and warm sub-humid regions of Nigeria, fresh fish consumed in rural villages are mostly sourced from inland fisheries.

We ran models with ten different spatial measures of forest cover to test the sensitivity of our model to variations in spatial scales. Results in S5 Table show that all measures of forest cover are significant and display very little differences between spatial bands. We observe higher significance and slightly larger coefficients of forest cover at closer proximities to villages (1km - 5km (v01-v05) buffer around villages, see S1 Fig).

To check the robustness of our results, we also ran the models on average processed fish consumption. We hypothesized that we would observe a weaker or insignificant relationship between the average consumption of processed fish and forests bordering rivers assuming that these types of fish are less likely to be locally captured than fresh fish. Results are found in Table 4. Consumption of processed fish did not have a significant association with forest cover around rivers across all forest cover measures (except for the spatial band *r1kmv01* but this relationship was negative and relatively weak). These results confirm our hypotheses that forest affect wild fish production, which in turn affects mainly fresh fish consumption since it is most likely sourced and consumed locally, unlike processed fish.

**Table 4:** Results from the second stage of the double hurdle model for processed fish consumption and forest cover around rivers at the village level across all measures of forest cover (see S6 Table for full results) Z-statistics are given in parentheses.

2^nd^ Stage	Processed fish intake			
*Forest cover* ª	*Coef*.	*Control variables*	*Pseudo R*^*2*^	*N*
r100v01	-0.002	Yes	0.340	309
	(-1.22)			
r100v05	-0.002	Yes	0.339	309
	(-0.83)			
r100v10	-0.001	Yes	0.338	309
	(-0.61)			
r500v01	-0.003	Yes	0.342	309
	(-1.49)			
r500v05	-0.003	Yes	0.340	309
	(-1.21)			
r500v10	-0.002	Yes	0.340	309
	(-1.05)			
r1kmv01	-0.003*	Yes	0.343	309
	(-1.65)			
r1kmv05	-0.003	Yes	0.341	309
	(-1.40)			
r1kmv10	-0.003	Yes	0.341	309
	(-1.27)			
r2kmv20	-0.003	Yes	0.341	309
	(-1.45)			

*p<0.1

**p<0.05

***p<0.01

ªRefer to Table 1 for descriptions of forest cover variables

Several studies have demonstrated that land cover within close proximity to river habitats critically influences in-stream habitats and aquatic fauna [61–63]. Other studies have assessed the links between land use and local diets, yet very few studies explore the relationship between spatial characteristics and fish consumption [64, 65] and none, to our knowledge, assess the relationship between forests and fish consumption. Our findings support the claim that forests can play a positive role in the productivity of freshwater fish populations, which is a vital source of protein for many rural households in Nigeria [66, 67].

However, there are some limitations to our study. First, we only categorized land cover into two classes (forest and non-forest) and therefore cannot assess how different forest types might affect fish consumption. Second, because of data constraints, we only captured fish consumption during one season; since fish can be seasonal and different species may have varied responses to forest cover, the impact of forest cover on fish consumption could differ in alternate seasons. Third, since the LSMS data do not have information on the source of the fish that were consumed, we could not be certain that the fresh fish consumed actually came from nearby rivers. We tried to address this by controlling for distance to coast, lakes, and nearest key market, but these controls are imperfect.

The data show that around 15% of households in our sample consumed fresh fish in rural areas in the week before the survey. This could indeed reflect the average amount of fish consumption in Nigeria during the survey period, however, other reasons could also explain the small sample of households consuming fresh fish. This could be due to the seasonal nature of the survey, or, the sampling method of the LSMS-ISA. Fishing communities tend to be clustered and isolated, which can result in their limited representativeness in national samples and an understatement of the importance of small scale fisheries in food security, particularly in rural regions [66].

## Conclusion

Understanding the links between the environmental landscape and food consumption patterns is important for assessing potential trade-offs between forest conservation and food security. For Nigeria, and many other developing countries, fish are a vital source of protein and micronutrients that help to sustain healthy diverse diets. However, the paucity of studies on forest and fish interactions in this region limits our understanding of the importance of forests for wild capture inland fisheries in this region. This study is a first attempt at using existing data sets to investigate whether or not there may be an important link worthy of attention. Here, we find a positive and statistically significant relationship between forests and freshwater fish consumption in Nigeria. This association is particularly strong in the humid and sub-humid regions of Nigeria where forest cover is relatively high. The results from our robustness test showing that this relationship only holds for fresh, and not processed fish, demonstrates that the relationship is not merely a statistical artefact, but indicative of a real relationship. Our findings emphasize the importance of forests for inland fisheries that deliver key sources of food to rural communities in Nigeria.

In order to deepen our understanding of these relationships, more detailed purposively collected data are necessary. Greater attention needs to be given to fishing communities in forest landscapes to better account for the importance of fish in rural livelihoods. Data that also targets fish production, seasonality, and fish processing can also enrich our understanding of the relationship between forests, inland fisheries, and food security.

The contribution of aquaculture to food production and diets is widely known and acknowledged; [68]; the importance of inland capture fisheries for local livelihoods and diets is, in comparison relatively under documented The research presented here points to forested rivers and floodplains as key contributors to supporting inland fisheries and communities that depend on wild-capture resources, and calls for greater attention on forest-freshwater ecosystems.

While there is a common perception that there exists a trade-off between forest conservation and food production, this idea implicitly equates food production with agriculture and most commonly with staple crop production [69]. Aquatic habitats tend to be overlooked in debates about land use and food production, and yet can be critically important sources of nutrient ric h foods that are limited in rural diets in developing countries, particularly for the poor. Research on the benefits and costs of deforestation to local communities has been widely documented (see for example Angelsen and Wunder [70] and Cheng, MacLeod [71]). The evidence presented here suggests that other additional consequences of deforestation also exist that have been relatively unexplored. Deforestation around rivers in particular, could negatively impact fish consumption in areas that are not well integrated into markets. Given the high rates of riparian and floodplain deforestation in Nigeria, a better understanding of what is gained and lost with these land use changes and the potential impacts on the nutrition and health of the communities living in these landscapes is critical.

## Supporting information

S1 Table Forest cover median and mean measured at varying spatial scales after excluding villages with zero forest cover. FC: Forest cover around rivers
(DOCX)

S2 Table Results of first stage hurdle model for forest cover *r1kmv01* and the decision to consume fresh fish for all rural villages and villages in warm-humid and warm sub-humid zones. **Z-statistics are given in parentheses.** *p<0.1 **p<0.05 ***p<0.01. AEZ: Agroecological zone
(DOCX)

S3 Table First and second stage of hurdle model for beef consumption. **Z-statistics are given in parentheses.** *p<0.1 **p<0.05 ***p<0.01. AEZ: Agroecological zone. ªForest cover within 100m width around river buffers and 1km radius around village.
(DOCX)

S4 Table First and second stage of hurdle model across all ten variables of forest cover representing different spatial measures in warm humid and warm sub-humid Agroecological Zones (AEZ) in Nigeria. **Z-statistics are given in parentheses.** *p<0.1 **p<0.05 ***p<0.01 ªRefer to Table 1 for descriptions of forest cover measures.
(DOCX)

S5 Table First and second stage of hurdle model for all ten measures of forest cover around rivers. **Z-statistics are given in parentheses.** *p<0.1 **p<0.05 ***p<0.01 ªRefer to Table 1 for descriptions of forest cover measures. AEZ: Agroecological zone.
(DOCX)

S6 Table First and second stage of hurdle model for processed fish intake (dried, frozen, and smoked) by households over seven day period. **Z-statistics are given in parentheses.** *p<0.1 **p<0.05 ***p<0.01 ªRefer to Table 1 for descriptions of forest cover measures. AEZ: Agroecological zone.
(DOCX)

S1 Fig Pseudo R^2^ and z-statistics for all measures of forest cover. See Table 1 for definitions of forest cover measure.
(DOCX)
