# Supplementary material for: The relationship between forests and freshwater fish consumption in rural Nigeria
Source: PLoS One. 2019 Jun 11;14(6):e0218038. doi: 10.1371/journal.pone.0218038 (PMC6559641; doi:10.1371/journal.pone.0218038)
Supplement: S1 Table — FC: Forest cover around rivers (DOCX) [file pone.0218038.s001.docx]

**S1 Table. Forest cover median and mean measured at varying spatial scales after excluding villages with zero forest cover. FC: Forest cover around rivers**

| **Statistics** | **r100v01** | **r100v05** | **r100v10** |  |
| --- | --- | --- | --- | --- |
| Av (FC>0) | 21.01 | 19.97 | 19.13 |  |
| Median(FC>0) | 12.16 | 11.29 | 10.12 |  |
|  | **r500v01** | **r500v05** | **r500v10** |  |
| Av (FC>0) | 19.81 | 19.30 | 18.51 |  |
| Median(FC>0) | 8.80 | 9.04 | 8.18 |  |
|  | **r1kmv01** | **r1kmv05** | **r1kmv10** | **r2kmv20** |
| Av (FC>0) | 19.28 | 18.63 | 18.33 | 16.80 |
| Median(FC>0) | 7.05 | 7.09 | 7.18 | 5.55 |
