# Supplementary material for: The relationship between forests and freshwater fish consumption in rural Nigeria
Source: PLoS One. 2019 Jun 11;14(6):e0218038. doi: 10.1371/journal.pone.0218038 (PMC6559641; doi:10.1371/journal.pone.0218038)
Supplement: S2 Table — Z-statistics are given in parentheses. *p<0.1 **p<0.05 ***p<0.01. AEZ: Agroecological zone (DOCX) [file pone.0218038.s002.docx]

**S2 Table. Results of first stage hurdle model for forest cover *r1kmv01* and the decision to consume fresh fish for all rural villages and villages in warm-humid and warm sub-humid zones. Z-statistics are given in parentheses.**

|  | **Fresh fish consumption (all rural villages)** | **Fresh fish consumption (Warm-humid regions only)** |
| --- | --- | --- |
| ***1^st^ Stage*** | ***I*** | ***II*** |
| Fresh fish price | 0.00025* | 0.0002 |
|  | (-1.67) | (-1.01) |
| Distance to lake | -0.008** | -0.014** |
|  | (-2.17) | (-2.55) |
| Distance to coast | 0.001 | 0.002** |
|  | (-1.22) | (-2.38) |
| Distance to market | -0.00008 | 0.003 |
|  | (-0.04) | (-1.15) |
| Elevation | -0.001 | -0.003*** |
|  | (-1.31) | (-3.26) |
| Warm-humid AEZ zone (dummy) | -0.061 |  |
|  | (-0.18) |  |
| Constant | -0.098 | -0.023 |
|  | (-0.22) | (-0.07) |
|  |  |  |
| **Pseudo R^2^** | **0.0873** | **0.192** |
| **N** | **309** | **190** |

*p<0.1 **p<0.05 ***p<0.01
AEZ: Agroecological zone
