# Supplementary material for: The relationship between forests and freshwater fish consumption in rural Nigeria
Source: PLoS One. 2019 Jun 11;14(6):e0218038. doi: 10.1371/journal.pone.0218038 (PMC6559641; doi:10.1371/journal.pone.0218038)
Supplement: S3 Table — Z-statistics are given in parentheses. *p<0.1 **p<0.05 ***p<0.01. AEZ: Agroecological zone. ªForest cover within 100m width around river buffers and 1km radius around village. (DOCX) [file pone.0218038.s003.docx]

**S3 Table. First and second stage of hurdle model for beef consumption. Z-statistics are given in parentheses.**

| 2^nd^ Stage | Coefficient |
| --- | --- |
| Forest cover (r100v01ª) | -0.002 |
|  | (-0.52) |
| Household size | 0.132*** |
|  | (3.21) |
| Age of household head | -0.010 |
|  | (-1.00) |
| Education of household head | -0.144 |
|  | (-0.93) |
| Wealth status of household | 0.236*** |
|  | (3.75) |
| Beef price | 0.0002 |
|  | (0.57) |
| Fresh fish price | -0.00031* |
|  | (-1.88) |
| Distance to lake | 0.000 |
|  | (0.01) |
| Distance to market | 0.000 |
|  | (0.26) |
| Distance to coast | 0.001 |
|  | (1.46) |
| Elevation | -0.0009** |
|  | (-2.27) |
| Constant | 0.047 |
|  | (0.05) |
| **1st Stage** |  |
| Beef price | 0.003*** |
|  | (4.17) |
| Distance to lake | 0.005 |
|  | (0.98) |
| Distance to coast | 0.000 |
|  | (-0.40) |
| Distance to market | 0.001 |
|  | (0.45) |
| Elevation | 0.00111* |
|  | (1.68) |
| Warm humid AEZ zone (dummy) | 0.423 |
|  | (0.84) |
| Constant | -1.231 |
|  | (-1.39) |
|  |  |
| **Pseudo R^2^** | **0.1916** |
| **N** | **309** |

*p<0.1 **p<0.05 ***p<0.01

AEZ: Agroecological zone

ªForest cover within 100m width around river buffers and 1km radius around village
