# Supplementary material for: The relationship between forests and freshwater fish consumption in rural Nigeria
Source: PLoS One. 2019 Jun 11;14(6):e0218038. doi: 10.1371/journal.pone.0218038 (PMC6559641; doi:10.1371/journal.pone.0218038)
Supplement: S4 Table — Z-statistics are given in parentheses. *p<0.1 **p<0.05 ***p<0.01 ªRefer to Table 1 for descriptions of forest cover measures. (DOCX) [file pone.0218038.s004.docx]

**S4A Table. Second stage of hurdle model across all ten variables of forest cover representing different spatial measures in warm humid and warm sub-humid Agroecological Zones (AEZ) in Nigeria. Z-statistics are given in parentheses.**

|  | **Forest cover measuresª** | | | |  |  |  |  |  |  |
| --- | --- | --- | --- | --- | --- | --- | --- | --- | --- | --- |
| **2^nd^ Stage** | **r100v01** | **r100v05** | **r100v10** | **r500v01** | **r500v05** | **r500v10** | **r1kmv01** | **r1kmv05** | **r1kmv10** | **r2kmv20** |
| Forest cover | 0.016*** | 0.018** | 0.018** | 0.016*** | 0.017*** | 0.017*** | 0.015*** | 0.017*** | 0.017*** | 0.016** |
|  | (2.59) | (2.55) | (2.42) | (2.68) | (2.71) | (2.56) | (2.73) | (2.78) | (2.59) | (2.42) |
| Household size | -0.047 | -0.036 | -0.044 | -0.059 | -0.047 | -0.051 | -0.057 | -0.048 | -0.050 | -0.057 |
|  | (-0.68) | (-0.52) | (-0.61) | (-0.86) | (-0.69) | (-0.72) | (-0.83) | (-0.70) | (-0.71) | (-0.79) |
| Age of household head | -0.005 | -0.003 | -0.004 | -0.002 | -0.001 | -0.002 | 0.0005 | 0.0005 | -0.001 | 0.0003 |
|  | (-0.30) | (-0.21) | (-0.21) | (-0.13) | (-0.07) | (-0.12) | (-0.03) | (0.03) | (-0.05) | (0.02) |
| Education of household head | 0.141 | 0.168 | 0.181 | 0.096 | 0.109 | 0.126 | 0.064 | 0.079 | 0.103 | 0.141 |
|  | (0.47) | (0.55) | (0.58) | (0.33) | (0.37) | (0.42) | (0.22) | (0.28) | (0.35) | (0.46) |
| Wealth index of household | -0.024 | -0.002 | 0.002 | -0.012 | 0.012 | 0.018 | 0.007 | 0.024 | 0.028 | 0.024 |
|  | (-0.25) | (-0.02) | (0.02) | (-0.13) | (0.13) | (0.19) | (0.07) | (0.26) | (0.29) | (0.25) |
| Beef consumed by household | -0.339 | -0.377 | -0.382 | -0.264 | -0.327 | -0.349 | -0.255 | -0.313 | -0.344 | -0.340 |
|  | (-1.04) | (-1.13) | (-1.11) | (-0.83) | (-1.02) | (-1.05) | (-0.82) | (-0.99) | (-1.05) | (-0.99) |
| Fresh fish price | 0.00003 | 0.00007 | 0.00011 | 0.00008 | 0.00009 | 0.00012 | 0.00010 | 0.00011 | 0.00014 | 0.00019 |
|  | (0.17) | (0.43) | (0.63) | (0.47) | (0.59) | (0.74) | (0.60) | (0.71) | (0.83) | (1.04) |
| Distance to lake | -0.013* | -0.011* | -0.011 | -0.011* | -0.010 | -0.009 | -0.010 | -0.008 | -0.008 | -0.008 |
|  | (-1.84) | (-1.69) | (-1.63) | (-1.72) | (-1.52) | (-1.43) | (-1.55) | (-1.36) | (-1.28) | (-1.26) |
| Distance to market | -0.004 | -0.005 | -0.006 | -0.005 | -0.005 | -0.006 | -0.005 | -0.005 | -0.006 | -0.006 |
|  | (-1.33) | (-1.48) | (-1.50) | (-1.48) | (-1.58) | (-1.57) | (-1.51) | (-1.62) | (-1.59) | (-1.52) |
| Distance to coast | 0.003** | 0.004** | 0.004** | 0.004** | 0.004** | 0.004** | 0.004** | 0.004** | 0.004** | 0.004** |
|  | (2.10) | (2.17) | (2.11) | (2.23) | (2.30) | (2.22) | (2.27) | (2.35) | (2.25) | (2.16) |
| Elevation | -0.003** | -0.003** | -0.003** | -0.003** | -0.003** | -0.003** | -0.003** | -0.003** | -0.003** | -0.003** |
|  | (-2.25) | (-2.28) | (-2.24) | (-2.25) | (-2.30) | (-2.25) | (-2.25) | (-2.29) | (-2.23) | (-2.14) |
| Constant | 0.436 | 0.132 | 0.099 | 0.258 | 0.037 | 0.029 | 0.148 | -0.057 | -0.051 | -0.236 |
|  | (0.33) | (0.10) | (0.07) | (0.20) | (0.03) | (0.02) | (0.11) | (-0.04) | (-0.04) | (-0.17) |
|  |  |  |  |  |  |  |  |  |  |  |
| **Pseudo R^2^** | **0.186** | **0.186** | **0.182** | **0.190** | **0.191** | **0.187** | **0.192** | **0.194** | **0.188** | **0.183** |
| **N** | **190** | **190** | **190** | **190** | **190** | **190** | **190** | **190** | **190** | **190** |

*p<0.1 **p<0.05 ***p<0.01

ªRefer to Table 1 for descriptions of forest cover measures

**S4B Table. Second stage of hurdle model across all ten variables of forest cover representing different spatial measures in warm humid and warm sub-humid Agroecological Zones (AEZ) in Nigeria. Z-statistics are given in parentheses.**

|  | **Forest cover measuresª** | | | |  |  |  |  |  |  |
| --- | --- | --- | --- | --- | --- | --- | --- | --- | --- | --- |
| **1^st^ Stage** | **r100v01** | **r100v05** | **r100v10** | **r500v01** | **r500v05** | **r500v10** | **r1kmv01** | **r1kmv05** | **r1kmv10** | **r2kmv20** |
| Fresh fish price | 0.0002 | 0.0002 | 0.0002 | 0.0002 | 0.0002 | 0.0002 | 0.0002 | 0.0002 | 0.0002 | 0.0002 |
|  | (1.01) | (1.01) | (1.01) | (1.01) | (1.01) | (1.01) | (1.01) | (1.01) | (1.01) | (1.01) |
| Distance to lake | -0.014** | -0.014** | -0.014** | -0.014** | -0.014** | -0.014** | -0.014** | -0.014** | -0.014** | -0.014** |
|  | (-2.55) | (-2.55) | (-2.55) | (-2.55) | (-2.55) | (-2.55) | (-2.55) | (-2.55) | (-2.55) | (-2.55) |
| Distance to coast | 0.002** | 0.002** | 0.002** | 0.002** | 0.002** | 0.002** | 0.002** | 0.002** | 0.002** | 0.002** |
|  | (2.38) | (2.38) | (2.38) | (2.38) | (2.38) | (2.38) | (2.38) | (2.38) | (2.38) | (2.38) |
| Distance to market | 0.003 | 0.003 | 0.003 | 0.003 | 0.003 | 0.003 | 0.003 | 0.003 | 0.003 | 0.003 |
|  | (1.15) | (1.15) | (1.15) | (1.15) | (1.15) | (1.15) | (1.15) | (1.15) | (1.15) | (1.15) |
| Elevation | -0.003*** | -0.003*** | -0.003*** | -0.003*** | -0.003*** | -0.003*** | -0.003*** | -0.003*** | -0.003*** | -0.003*** |
|  | (-3.26) | (-3.26) | (-3.26) | (-3.26) | (-3.26) | (-3.26) | (-3.26) | (-3.26) | (-3.26) | (-3.26) |
| constant | -0.023 | -0.023 | -0.023 | -0.023 | -0.023 | -0.023 | -0.023 | -0.023 | -0.023 | -0.023 |
|  | (-0.07) | (-0.07) | (-0.07) | (-0.07) | (-0.07) | (-0.07) | (-0.07) | (-0.07) | (-0.07) | (-0.07) |
|  |  |  |  |  |  |  |  |  |  |  |
| **Pseudo R^2^** | **0.186** | **0.186** | **0.182** | **0.190** | **0.191** | **0.187** | **0.192** | **0.194** | **0.188** | **0.183** |
| **N** | **190** | **190** | **190** | **190** | **190** | **190** | **190** | **190** | **190** | **190** |

*p<0.1 **p<0.05 ***p<0.01

ªRefer to Table 1 for descriptions of forest cover measure
