# Supplementary material for: The relationship between forests and freshwater fish consumption in rural Nigeria
Source: PLoS One. 2019 Jun 11;14(6):e0218038. doi: 10.1371/journal.pone.0218038 (PMC6559641; doi:10.1371/journal.pone.0218038)
Supplement: S5 Table — Z-statistics are given in parentheses. *p<0.1 **p<0.05 ***p<0.01 ªRefer to Table 1 for descriptions of forest cover measures. AEZ: Agroecological zone. (DOCX) [file pone.0218038.s005.docx]

**S5A Table. Results from the second stage of hurdle model for all ten measures of forest cover around rivers. Z-statistics are given in parentheses.**

|  | **Forest cover measuresª** | | | |  | |  | |  |  |  |  |
| --- | --- | --- | --- | --- | --- | --- | --- | --- | --- | --- | --- | --- |
| **2^nd^ Stage** | **r100v01** | **r100v05** | **r100v10** | **r500v01** | | **r500v05** | | **r500v10** | **r1kmv01** | **r1kmv05** | **r1kmv10** | **r2kmv20** |
| Forest cover | 0.017* | 0.017* | 0.016* | 0.016* | | 0.016* | | 0.015* | 0.015* | 0.015* | 0.015* | 0.014* |
|  | (1.86) | (1.82) | (1.67) | (1.90) | | (1.90) | | (1.78) | (1.94) | (1.95) | (1.81) | (1.68) |
| Household size | 0.098 | 0.107 | 0.102 | 0.091 | | 0.101 | | 0.100 | 0.093 | 0.100 | 0.100 | 0.096 |
|  | (1.05) | (1.12) | (1.06) | (1.00) | | (1.09) | | (1.06) | (1.02) | (1.09) | (1.07) | (1.01) |
| Age of household head | -0.019 | -0.018 | -0.018 | -0.015 | | -0.014 | | -0.015 | -0.013 | -0.012 | -0.014 | -0.013 |
|  | (-0.81) | (-0.77) | (-0.75) | (-0.65) | | (-0.64) | | (-0.66) | (-0.57) | (-0.56) | (-0.60) | (-0.56) |
| Education of household head | -0.116 | -0.114 | -0.111 | -0.134 | | -0.136 | | -0.131 | -0.152 | -0.150 | -0.142 | -0.129 |
|  | (-0.33) | (-0.32) | (-0.31) | (-0.38) | | (-0.39) | | (-0.37) | (-0.44) | (-0.43) | (-0.40) | (-0.36) |
| Wealth index of household | 0.021 | 0.042 | 0.049 | 0.027 | | 0.045 | | 0.052 | 0.038 | 0.051 | 0.056 | 0.058 |
|  | (0.16) | (0.32) | (0.36) | (0.20) | | (0.35) | | (0.39) | (0.29) | (0.39) | (0.43) | (0.43) |
| Beef consumed by household | -0.184 | -0.193 | -0.198 | -0.142 | | -0.164 | | -0.177 | -0.138 | -0.156 | -0.173 | -0.172 |
|  | (-0.55) | (-0.57) | (-0.57) | (-0.43) | | (-0.49) | | (-0.52) | (-0.42) | (-0.47) | (-0.51) | (-0.50) |
| Fresh fish price | 0.0004 | 0.0004 | 0.0004 | 0.0004 | | 0.0004 | | 0.0004 | 0.0004 | 0.0004 | 0.0004 | 0.0005 |
|  | (1.20) | (1.29) | (1.34) | (1.32) | | (1.36) | | (1.39) | (1.37) | (1.40) | (1.42) | (1.46) |
| Distance to lake | -0.026** | -0.025** | -0.025** | -0.025** | | -0.024** | | -0.024** | -0.024** | -0.023** | -0.024** | -0.024** |
|  | (-2.16) | (-2.13) | (-2.10) | (-2.16) | | (-2.13) | | (-2.10) | (-2.15) | (-2.12) | (-2.09) | (-2.07) |
| Distance to market | -0.0005 | -0.0008 | -0.0010 | -0.0005 | | -0.0006 | | -0.0007 | -0.0004 | -0.0005 | -0.0007 | -0.0006 |
|  | (-0.14) | (-0.23) | (-0.27) | (-0.15) | | (-0.17) | | (-0.21) | (-0.13) | (-0.15) | (-0.19) | (-0.18) |
| Distance to coast | 0.001 | 0.001 | 0.001 | 0.001 | | 0.001 | | 0.001 | 0.001 | 0.001 | 0.001 | 0.002 |
|  | (1.48) | (1.51) | (1.48) | (1.53) | | (1.56) | | (1.53) | (1.55) | (1.59) | (1.55) | (1.52) |
| Elevation | -0.002* | -0.002* | -0.002* | -0.002* | | -0.002* | | -0.002* | -0.002* | -0.002* | -0.002* | -0.002* |
|  | (-1.82) | (-1.83) | (-1.81) | (-1.77) | | (-1.79) | | (-1.78) | (-1.76) | (-1.77) | (-1.76) | (-1.73) |
| Constant | 0.125 | -0.033 | -0.029 | -0.118 | | -0.219 | | -0.187 | -0.235 | -0.330 | -0.279 | -0.355 |
|  | (0.07) | (-0.02) | (-0.02) | (-0.07) | | (-0.13) | | (-0.10) | (-0.13) | (-0.19) | (-0.16) | (-0.19) |
|  |  |  |  |  | |  | |  |  |  |  |  |
| **Pseudo R^2^** | **0.0861** | **0.0853** | **0.0835** | **0.0867** | | **0.0865** | | **0.0848** | **0.0873** | **0.0871** | **0.0853** | **0.0838** |
| **N** | **309** | **309** | **309** | **309** | | **309** | | **309** | **309** | **309** | **309** | **309** |

*p<0.1 **p<0.05 ***p<0.01

**ª**Refer to Table 1 for descriptions of forest cover measures

**S5B Table. Results from the first stage of hurdle model for all ten measures of forest cover around rivers. Z-statistics are given in parentheses.**

|  | **Forest cover measuresª** | | | |  |  |  |  |  |  |
| --- | --- | --- | --- | --- | --- | --- | --- | --- | --- | --- |
| **1^st^ Stage** | **r100v01** | **r100v05** | **r100v10** | **r500v01** | **r500v05** | **r500v10** | **r1kmv01** | **r1kmv05** | **r1kmv10** | **r2kmv20** |
| Fresh fish price | 0.00025* | 0.00025* | 0.00025* | 0.00025* | 0.00025* | 0.00025* | 0.00025* | 0.00025* | 0.00025* | 0.00025* |
|  | (1.67) | (1.67) | (1.67) | (1.67) | (1.67) | (1.67) | (1.67) | (1.67) | (1.67) | (1.67) |
| Distance to lake | -0.008** | -0.008** | -0.008** | -0.008** | -0.008** | -0.008** | -0.008** | -0.008** | -0.008** | -0.008** |
|  | (-2.17) | (-2.17) | (-2.17) | (-2.17) | (-2.17) | (-2.17) | (-2.17) | (-2.17) | (-2.17) | (-2.17) |
| Distance to coast | 0.001 | 0.001 | 0.001 | 0.001 | 0.001 | 0.001 | 0.001 | 0.001 | 0.001 | 0.001 |
|  | (1.22) | (1.22) | (1.22) | (1.22) | (1.22) | (1.22) | (1.22) | (1.22) | (1.22) | (1.22) |
| Distance to market | -0.00008 | -0.00008 | -0.00008 | -0.00008 | -0.00008 | -0.00008 | -0.00008 | -0.00008 | -0.00008 | -0.00008 |
|  | (-0.04) | (-0.04) | (-0.04) | (-0.04) | (-0.04) | (-0.04) | (-0.04) | (-0.04) | (-0.04) | (-0.04) |
| Elevation | -0.001 | -0.001 | -0.001 | -0.001 | -0.001 | -0.001 | -0.001 | -0.001 | -0.001 | -0.001 |
|  | (-1.31) | (-1.31) | (-1.31) | (-1.31) | (-1.31) | (-1.31) | (-1.31) | (-1.31) | (-1.31) | (-1.31) |
| warm-humid AEZ zone (dummy) | -0.061 | -0.061 | -0.061 | -0.061 | -0.061 | -0.061 | -0.061 | -0.061 | -0.061 | -0.061 |
|  | (-0.18) | (-0.18) | (-0.18) | (-0.18) | (-0.18) | (-0.18) | (-0.18) | (-0.18) | (-0.18) | (-0.18) |
| constant | -0.098 | -0.098 | -0.098 | -0.098 | -0.098 | -0.098 | -0.098 | -0.098 | -0.098 | -0.098 |
|  | (-0.22) | (-0.22) | (-0.22) | (-0.22) | (-0.22) | (-0.22) | (-0.22) | (-0.22) | (-0.22) | (-0.22) |
|  |  |  |  |  |  |  |  |  |  |  |
| **Pseudo R^2^** | **0.0861** | **0.0853** | **0.0835** | **0.0867** | **0.0865** | **0.0848** | **0.0873** | **0.0871** | **0.0853** | **0.0838** |
| **N** | **309** | **309** | **309** | **309** | **309** | **309** | **309** | **309** | **309** | **309** |

*p<0.1 **p<0.05 ***p<0.01

**ª**Refer to Table 1 for descriptions of forest cover measures

AEZ: Agroecological zone
