# Supplementary material for: The relationship between forests and freshwater fish consumption in rural Nigeria
Source: PLoS One. 2019 Jun 11;14(6):e0218038. doi: 10.1371/journal.pone.0218038 (PMC6559641; doi:10.1371/journal.pone.0218038)
Supplement: S6 Table — Z-statistics are given in parentheses. *p<0.1 **p<0.05 ***p<0.01 ªRefer to Table 1 for descriptions of forest cover measures. AEZ: Agroecological zone. (DOCX) [file pone.0218038.s006.docx]

**S6A Table. Second stage of hurdle model for processed fish intake (dried, frozen, and smoked) by households over seven day period. Z-statistics are given in parentheses.**

|  | **Forest cover measuresª** | | | |  |  |  |  |  |  |
| --- | --- | --- | --- | --- | --- | --- | --- | --- | --- | --- |
| **2^nd^ Stage** | **r100v01** | **r100v05** | **r100v10** | **r500v01** | **r500v05** | **r500v10** | **r1kmv01** | **r1kmv05** | **r1kmv10** | **r2kmv20** |
| Forest cover | -0.002 | -0.002 | -0.001 | -0.003 | -0.003 | -0.002 | -0.003* | -0.003 | -0.003 | -0.003 |
|  | (-1.22) | (-0.83) | (-0.61) | (-1.49) | (-1.21) | (-1.05) | (-1.65) | (-1.40) | (-1.27) | (-1.45) |
| Household size | 0.083*** | 0.085*** | 0.086*** | 0.083*** | 0.083*** | 0.084*** | 0.083*** | 0.083*** | 0.083*** | 0.083*** |
|  | (2.82) | (2.85) | (2.88) | (2.84) | (2.83) | (2.85) | (2.84) | (2.83) | (2.83) | (2.82) |
| Age of household head | 0.001 | 0.001 | 0.001 | 0.001 | 0.001 | 0.001 | 0.000 | 0.000 | 0.000 | 0.000 |
|  | (0.16) | (0.14) | (0.12) | (0.11) | (0.11) | (0.11) | (0.08) | (0.07) | (0.08) | (0.01) |
| Education of household head | -0.124 | -0.128 | -0.130 | -0.119 | -0.121 | -0.122 | -0.114 | -0.117 | -0.117 | -0.114 |
|  | (-1.05) | (-1.08) | (-1.10) | (-1.01) | (-1.03) | (-1.03) | (-0.98) | (-0.99) | (-0.99) | (-0.96) |
| Wealth index of household | 0.100** | 0.100** | 0.100** | 0.100** | 0.099** | 0.098** | 0.099** | 0.098** | 0.097** | 0.097** |
|  | (2.34) | (2.32) | (2.32) | (2.34) | (2.31) | (2.29) | (2.32) | (2.29) | (2.27) | (2.26) |
| Beef consumed by household | 0.068 | 0.067 | 0.067 | 0.066 | 0.068 | 0.070 | 0.065 | 0.068 | 0.070 | 0.069 |
|  | (0.59) | (0.58) | (0.58) | (0.57) | (0.58) | (0.60) | (0.57) | (0.58) | (0.60) | (0.59) |
| Fresh fish price | 0.00003 | 0.00004 | 0.00004 | 0.00003 | 0.00003 | 0.00003 | 0.00003 | 0.00003 | 0.00003 | 0.00002 |
|  | (0.43) | (0.46) | (0.47) | (0.40) | (0.42) | (0.41) | (0.37) | (0.39) | (0.37) | (0.30) |
| Distance to lake | 0.004** | 0.004** | 0.004** | 0.004** | 0.004** | 0.004** | 0.004** | 0.004** | 0.004** | 0.004* |
|  | (2.09) | (2.07) | (2.08) | (2.05) | (2.03) | (2.02) | (2.01) | (1.99) | (1.98) | (1.94) |
| Distance to market | -0.00020 | -0.00021 | -0.00023 | -0.00020 | -0.00020 | -0.00020 | -0.00020 | -0.00019 | -0.00019 | -0.00021 |
|  | (-0.18) | (-0.19) | (-0.20) | (-0.17) | (-0.18) | (-0.18) | (-0.18) | (-0.17) | (-0.17) | (-0.19) |
| Distance to coast | -0.002*** | -0.002*** | -0.002*** | -0.002*** | -0.002*** | -0.002*** | -0.002*** | -0.002*** | -0.002*** | -0.002*** |
|  | (-4.85) | (-4.74) | (-4.66) | (-4.92) | (-4.84) | (-4.78) | (-4.96) | (-4.89) | (-4.85) | (-4.88) |
| Elevation | 0.000 | 0.000 | 0.000 | 0.000 | 0.000 | 0.000 | 0.000 | 0.000 | 0.000 | 0.000 |
|  | (-0.18) | (-0.18) | (-0.19) | (-0.22) | (-0.21) | (-0.21) | (-0.24) | (-0.23) | (-0.23) | (-0.26) |
| Constant | 0.568 | 0.554 | 0.545 | 0.597 | 0.588 | 0.579 | 0.620 | 0.613 | 0.605 | 0.654 |
|  | (0.99) | (0.96) | (0.95) | (1.04) | (1.02) | (1.01) | (1.08) | (1.07) | (1.05) | (1.13) |
|  |  |  |  |  |  |  |  |  |  |  |
| **Pseudo R^2^** | **0.340** | **0.339** | **0.338** | **0.342** | **0.340** | **0.340** | **0.343** | **0.341** | **0.341** | **0.341** |
| **N** | **309** | **309** | **309** | **309** | **309** | **309** | **309** | **309** | **309** | **309** |

*p<0.1 **p<0.05 ***p<0.01
ªRefer to Table 1 for descriptions of forest cover measures
AEZ: Agroecological Zone

**S6B Table. First stage of hurdle model for processed fish intake (dried, frozen, and smoked) by households over seven day period. Z-statistics are given in parentheses.**

|  | **Forest cover measuresª** | | | |  |  |  |  |  |  |
| --- | --- | --- | --- | --- | --- | --- | --- | --- | --- | --- |
| **1^st^ Stage** | **r100v01** | **r100v05** | **r100v10** | **r500v01** | **r500v05** | **r500v10** | **r1kmv01** | **r1kmv05** | **r1kmv10** | **r2kmv20** |
| Fresh fish price | 0.00033 | 0.00033 | 0.00033 | 0.00033 | 0.00033 | 0.00033 | 0.00033 | 0.00033 | 0.00033 | 0.00033 |
|  | (0.60) | (0.60) | (0.60) | (0.60) | (0.60) | (0.60) | (0.60) | (0.60) | (0.60) | (0.60) |
| Distance to lake | 0.006 | 0.006 | 0.006 | 0.006 | 0.006 | 0.006 | 0.006 | 0.006 | 0.006 | 0.006 |
|  | (1.10) | (1.10) | (1.10) | (1.10) | (1.10) | (1.10) | (1.10) | (1.10) | (1.10) | (1.10) |
| Distance to coast | -0.001 | -0.001 | -0.001 | -0.001 | -0.001 | -0.001 | -0.001 | -0.001 | -0.001 | -0.001 |
|  | (-0.78) | (-0.78) | (-0.78) | (-0.78) | (-0.78) | (-0.78) | (-0.78) | (-0.78) | (-0.78) | (-0.78) |
| Distance to market | 0.001 | 0.001 | 0.001 | 0.001 | 0.001 | 0.001 | 0.001 | 0.001 | 0.001 | 0.001 |
|  | (0.34) | (0.34) | (0.34) | (0.34) | (0.34) | (0.34) | (0.34) | (0.34) | (0.34) | (0.34) |
| Elevation | -0.00025 | -0.00025 | -0.00025 | -0.00025 | -0.00025 | -0.00025 | -0.00025 | -0.00025 | -0.00025 | -0.00025 |
|  | (-0.40) | (-0.40) | (-0.40) | (-0.40) | (-0.40) | (-0.40) | (-0.40) | (-0.40) | (-0.40) | (-0.40) |
| Warm humid AEZ zone (dummy) | 1.585** | 1.585** | 1.585** | 1.585** | 1.585** | 1.585** | 1.585** | 1.585** | 1.585** | 1.585** |
|  | (2.45) | (2.45) | (2.45) | (2.45) | (2.45) | (2.45) | (2.45) | (2.45) | (2.45) | (2.45) |
| Constant | 0.748 | 0.748 | 0.748 | 0.748 | 0.748 | 0.748 | 0.748 | 0.748 | 0.748 | 0.748 |
|  | (0.72) | (0.72) | (0.72) | (0.72) | (0.72) | (0.72) | (0.72) | (0.72) | (0.72) | (0.72) |
|  |  |  |  |  |  |  |  |  |  |  |
| **Pseudo R^2^** | **0.340** | **0.339** | **0.338** | **0.342** | **0.340** | **0.340** | **0.343** | **0.341** | **0.341** | **0.341** |
| **N** | **309** | **309** | **309** | **309** | **309** | **309** | **309** | **309** | **309** | **309** |

*p<0.1 **p<0.05 ***p<0.01
ªRefer to Table 1 for descriptions of forest cover measures
AEZ: Agroecological Zone
