# Supplementary material for: The relationship between forests and freshwater fish consumption in rural Nigeria
Source: PLoS One. 2019 Jun 11;14(6):e0218038. doi: 10.1371/journal.pone.0218038 (PMC6559641; doi:10.1371/journal.pone.0218038)
Supplement: S1 Fig — See Table 1 for definitions of forest cover measure. (DOCX) [file pone.0218038.s007.docx]

**S1 Fig. Pseudo R^2^ and z-statistics for all measures of forest cover. See Table 1 for definition of forest cover measure.**
